# Use of hamstring autografts for ACL reconstruction significantly decreased the risk of reporting problems with kneeling at a 5‐year follow‐up

**DOI:** 10.1002/jeo2.70577

**Published:** 2025-11-28

**Authors:** Filip Vuletić, Eivind Inderhaug, R. Kyle Martin, Jon Olav Drogset, Ann Kristin Hansen, Stein Håkon Låstad Lygre, Håvard Visnes, Andreas Persson

**Affiliations:** ^1^ Department of Molecular Medicine and Surgery, Stockholm Sports Trauma Research Center Karolinska Institutet, Solna Stockholm Sweden; ^2^ Department of Arthroscopy and Sports Medicine, Orthopaedic Department Aker Oslo University Hospital Oslo Norway; ^3^ Haukeland University Hospital Bergen Norway; ^4^ Department of Clinical Medicine University of Bergen Bergen Norway; ^5^ Department of Orthopedic Surgery University of Minnesota Minneapolis Minnesota USA; ^6^ Department of Orthopedic Surgery Centracare M Physicians Orthopedics Saint Cloud Minnesota USA; ^7^ Oslo Sports Trauma Research Center Norwegian School of Sport Sciences Oslo Norway; ^8^ Trondheim University Hospital and Norwegian University of Science and Technology Trondheim Norway; ^9^ Department of Orthopaedic Surgery University Hospital of North Norway Tromsø Norway; ^10^ Institute of Clinical Medicine, Faculty of Health Sciences UiT‐ The Arctic University of Norway Tromsø Norway; ^11^ Department of Occupational Medicine Haukeland University Hospital Bergen Norway; ^12^ Department of Orthopedic Surgery, The Norwegian Knee Ligament Register Haukeland University Hospital Bergen Norway; ^13^ Department of Orthopedics Sorlandet Hospital Kristiansand Norway

**Keywords:** anterior cruciate ligament reconstruction, hamstring tendon autograft, bone–patellar tendon–bone autograft, kneeling difficulty, long‐term follow‐up

## Abstract

**Purpose:**

To compare 5‐year patient‐reported outcomes between hamstring tendon (HT) and bone–patellar tendon–bone (BPTB) autografts in primary anterior cruciate ligament reconstruction (ACLR), with particular focus on kneeling difficulties, extension deficits, and other functional limitations that have not been examined in long‐term follow‐up studies.

**Methods:**

This retrospective analysis of prospectively collected data from the Norwegian Knee Ligament Register included 10,329 patients who underwent primary ACLR between 2004 and 2017 using either HT (*n* = 6650) or BPTB (*n* = 3679) autografts with 5‐year follow‐up. Primary outcomes were assessed using specific KOOS questions: P1 (knee pain frequency), S4 (knee extension ability), and SP5 (kneeling difficulty). Secondary outcomes included achieving Patient‐Acceptable Symptom State (PASS) thresholds for KOOS Sport/Recreation ( ≥ 75.0) and Quality of Life ( ≥ 62.5) subscales. Multivariable logistic regression analyses were performed to identify factors associated with treatment success or failure, adjusting for age, sex, activity level, cartilage injury, meniscal injury, and time from injury to surgery.

**Results:**

Among 10,329 patients available for evaluation from 19,564 eligible procedures (53% follow‐up response rate), HT grafts demonstrated a significantly reduced risk of kneeling difficulties (SP5) at 5‐year follow‐up compared to BPTB grafts (OR 0.57, 95% CI 0.51–0.62, *p* < 0.001). HT recipients had higher odds of achieving PASS for the Sport/Recreation subscale (OR 1.29, 95% CI 1.19–1.41, *p* < 0.001), but showed no difference in achieving PASS for Quality of Life. Risk factors for worse outcomes included female sex, cartilage injury, delayed surgery ( > 12 months), and participation in pivoting sports at injury.

**Conclusions:**

This represents the first 5‐year comparative study examining graft‐specific differences in kneeling difficulties and extension deficits following ACLR. HT autografts significantly reduced the risk of kneeling problems and improved KOOS Sport/Recreation outcomes compared to BPTB grafts, while showing comparable results for pain and extension. These findings provide evidence for personalised discussions about graft selection.

**Level of Evidence:**

Level III.

## Graphical abstract

Hamstring autografts reduced kneeling difficulties by 43% compared to bone‐patellar tendon‐bone grafts at 5‐year follow‐up after ACL reconstruction in 10,329 patients from a national registry.

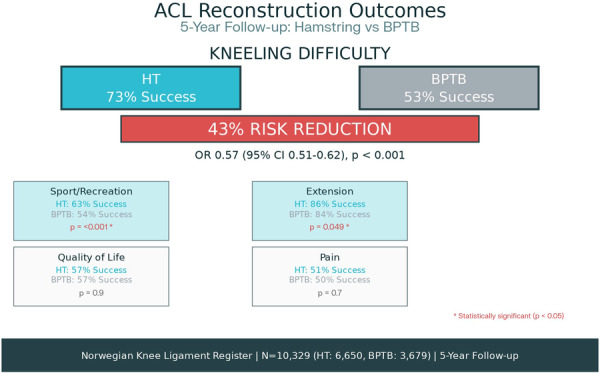

**Abbreviations**
ACL: anterior cruciate ligament
ACLR: anterior cruciate ligament reconstruction
BMI: body mass index
BPTB: bone–patellar tendon–bone
CI: confidence interval
HT: hamstring tendons
IQR: interquartile range
KOOS: Knee Injury and Osteoarthritis Outcome Score
NKLR: Norwegian Knee Ligament Register
OR: odds ratio
PASS: Patient‐Acceptable Symptom State
PROM: Patient Reported Outcome Measure
QoL: quality of life
Sports/Rec: sports/recreation

## INTRODUCTION

Anterior cruciate ligament (ACL) injuries are common and serious knee injuries in athletes, with an incidence of 68.6–81.1 injuries per 100,000 people per year [34]. They often occur during sporting activities and can lead to knee instability, pain, and restricted physical activity [8]. Untreated unstable knees following ACL tears can accelerate joint degeneration and may lead to osteoarthritis within 10–15 years, highlighting the importance of effective surgical intervention [38]. The choice of graft for ACL reconstruction is critical, with the most common options being hamstring tendons (HT) and bone–patellar tendon–bone (BPTB) autografts [32]. In Norway, about 2000 ACL reconstructions (ACLR) are performed each year. Before 2013, HT was the most common graft choice for ACLR in Norway. However, after finding that HT grafts had a higher revision rate compared to BPTB grafts, the trend reversed, and BPTB became the most common graft choice [8, 24]. A range of publications suggests that clinical and functional outcomes are comparable; both surgeon preferences and patient characteristics often influence the individual graft choice [32].

When discussing the options with patients, the morbidity associated with each graft choice is often discussed to enable a more informed decision. The most common concerns regarding BPTB are increased anterior knee pain and loss of extension, which may impact daily activities [3, 18, 21]. In contrast, HT autografts may result in hamstring weakness and potential flexion impairment [22, 25]. Longitudinal studies suggest that anterior knee pain may decrease over time, indicating potential symptom resolution with extended follow‐up [18, 21].

The Knee Injury and Osteoarthritis Outcome Score (KOOS) is a validated Patient Reported Outcome Measure (PROM) commonly used following ACLR [4]. Using 42 questions, it measures five dimensions: Pain, Symptoms, Activities of Daily Living, Sport and Recreation, and Knee‐related Quality of Life (QoL) [30]. The Patient‐Acceptable Symptom State (PASS) provides a patient‐centred benchmark based on individuals' perceptions of acceptable symptom levels [36]. Incorporating PASS helps interpret PROM scores, enabling us to determine whether patients perceive they have achieved a meaningful recovery [36].

Despite the widespread use of both HT and BPTB in ACL reconstruction, critical long‐term evidence differentiating their long‐term patient‐reported outcomes, especially regarding anterior knee pain and kneeling function, remains sparse. Prior research has largely focused on revision rates and general functional recovery, with less attention to specific patient experiences and real‐world limitations relevant to graft choice. Furthermore, the integration of patient‐centred metrics such as the PASS is still limited, leaving key benchmarks for meaningful clinical success underexplored. By providing the most extensive national registry‐based analysis of KOOS outcomes over five years, focusing on kneeling difficulty and extension deficits, as well as PASS outcomes related to activity satisfaction, this study fills a significant gap in the literature. These findings are poised to inform the nuanced, shared decision‐making now expected in contemporary ACL care and may shape future recommendations for personalised graft selection in both general and athletic populations.

This study compared five‐year patient‐reported outcomes between HT grafts and BPTB autografts in primary ACL reconstruction. The focus was on donor‐site morbidity, specifically kneeling difficulties and extension deficits. The primary hypothesis was that hamstring grafts would yield better outcomes for kneeling function due to less anterior knee pain and fewer donor‐site complications. Additionally, it was hypothesised that patients with HT grafts would have higher rates of achieving acceptable symptom levels in sports and recreational activities compared to those who received BPTB grafts.

## MATERIALS AND METHODS

This retrospective registry‐based analysis of prospectively collected data from the Norwegian Knee Ligament Register was conducted in accordance with established guidelines for registry‐based studies and reported in accordance with the Strengthening the Reporting of Observational Studies in Epidemiology (STROBE) statement [7].

### The Norwegian Knee Ligament Register (NKLR)

The NKLR was established in 2004 and aims to improve understanding and treatment of knee ligament injuries [10]. It collects detailed data on cruciate ligament surgeries and subsequent knee operations, and since 2017, surgeons report this information after surgery [17]. Surgeons submit patient information, intraoperative findings, and surgical techniques immediately after surgery. Patient‐reported outcomes are collected using the KOOS at baseline and at 2‐, 5‐ and 10‐year post‐surgery follow‐ups.

### Patient population

All patients who underwent primary anterior cruciate ligament reconstruction (ACLR) between 2004 and 2017 and were registered in NKLR (*n* = 23,486) with a 5‐year follow‐up were eligible for inclusion. Inclusion criteria included primary ACLR performed using either an HT or PT autograft. Systematic exclusion criteria were applied to ensure homogeneity: revision ACLR within 5 years after surgery (*n* = 1078), use of graft types other than HT or BPTB autografts (*n* = 693), concomitant ligament injuries (*n* = 2094; including posterior cruciate ligament (*n* = 311), medial collateral ligament (*n* = 1666), lateral collateral ligament (*n* = 369), and posterolateral corner (*n* = 195)), and other associated injuries (*n* = 57; one popliteal artery injury, three nerve damages, and 53 fractures of the femur (14), tibia (32), fibula (2), and others (5)).

### Definitions of KOOS outcomes

KOOS scoring followed established guidelines, with scores ranging from 0 to 100, where higher scores indicate superior outcomes [30]. Each subscale is evaluated separately.

Three clinically relevant primary outcome questions were selected based on their relevance to donor‐site morbidity and functional limitations: *P1 (“How often do you experience knee pain?”)*, assessing discomfort; *S4 (“Can you fully straighten your knee?”)*, indicating extension deficits; and *SP5 (“What difficulty have you had kneeling in the last week?”)*, reflecting functional limitations.

The primary outcome questions were defined in terms of success or failure as follows:

For P1, treatment success was defined as a score of 0–2 (never, monthly, weekly); scores of 3 and 4 (daily or constant pain) were considered treatment failures.

For S4, treatment success was defined as a score of 0–3 (always, often, sometimes, rarely); a score of 4 (indicating ‘never’) was considered a treatment failure.

For SP5, treatment success was defined as scores of 0–2 (none, mild, moderate), while scores of 3 and 4 (severe and extreme pain) were considered treatment failures [27].

The S4 classification differs from P1 and SP5 because the KOOS S4 question assesses knee extension ability using a reverse‐scoring system, where 4 indicates ‘never’ being able to straighten the knee fully [4, 31]. Clinical consensus defines any limitation in full extension (score of 4) as functionally significant failure, while occasional limitations (scores 0–3: ‘always,’ ‘often,’ ‘sometimes,’ ‘rarely’) are considered acceptable outcomes that do not preclude functional activities. This differs from the P1 and SP5 questions where scores of 3–4 represent daily/constant symptoms requiring clinical attention. Therefore, for S4, we classified scores 0–3 as clinical success and score 4 as failure, maintaining consistency with the established clinical interpretation of extension deficits in ACL reconstruction outcomes.

As a secondary outcome measure, we compared the number and proportion of patients achieving the Patient Acceptable Symptom State (PASS) at the 5‐year follow‐up between the two types of grafts. Established cut‐off values were used after ACLR, the KOOS Sports/Recreation (Sports/Rec) score of 75.0 (sensitivity: 0.87, specificity: 0.88), and the KOOS QoL score of 62.5 (sensitivity: 0.82, specificity: 0.85) [21].

The subscales Sport/Rec and QoL at the 5‐year follow‐up were compared between two graft types, while adjusting for clinically relevant factors (age, sex, activity level at the time of injury, cartilage injury, meniscal injury, BMI, and time from injury to surgery).

### Covariates and risk stratification

Comprehensive risk stratification was performed using clinically relevant variables: graft type, sex, age ( ≤ 25 years, >25 years), time to surgery ( ≤ 1 year,> 1 year), body mass index (BMI; kg/m²) ( < 20, 20–29, >30), and intraoperative findings including cartilage injury (yes/no) and meniscal injury (yes/no, medial and/or lateral). Activity classification followed established sports medicine categories: “pivoting sports” (soccer, handball, floorball, basketball), “other sports” (running, cycling, swimming), and “other activities,” recognising the differential risk profiles associated with specific athletic demands.

### Statistics

Descriptive statistics were presented as means with standard deviations or medians with interquartile ranges as appropriate. Between‐group comparisons utilised the proper statistical tests: the Mann–Whitney *U* test for continuous variables and the Pearson chi‐square test for categorical variables.

Multivariable logistic regression analysis was performed to identify independent risk factors associated with treatment success/failure for the three primary outcome questions (P1, S4, SP5), with results presented as odds ratios with 95% confidence intervals. Similar regression modelling identified factors associated with achieving PASS thresholds for KOOS Sport/Recreation and QoL subscales. Additionally, multiple linear regression analysis quantified the influence of patient and surgical factors on absolute 5‐year postoperative KOOS values for the Sport/Recreation and QoL subscales, adjusting for covariates including graft type, age, sex, time to surgery, activity type, and concurrent cartilage and meniscal injuries.

BMI data were missing in 37% of cases and were excluded from primary analyses but included in the responder/non‐responder analysis. Patients lacking complete data for the three primary KOOS outcome questions (P1, S4, SP5) were included only in the responder/non‐responder analysis (*n* = 9,317). Statistical analyses were conducted using IBM SPSS Statistics version 29.0, with significance set at *p* < 0.05 (two‐tailed).

## RESULTS

**Figure 1:**
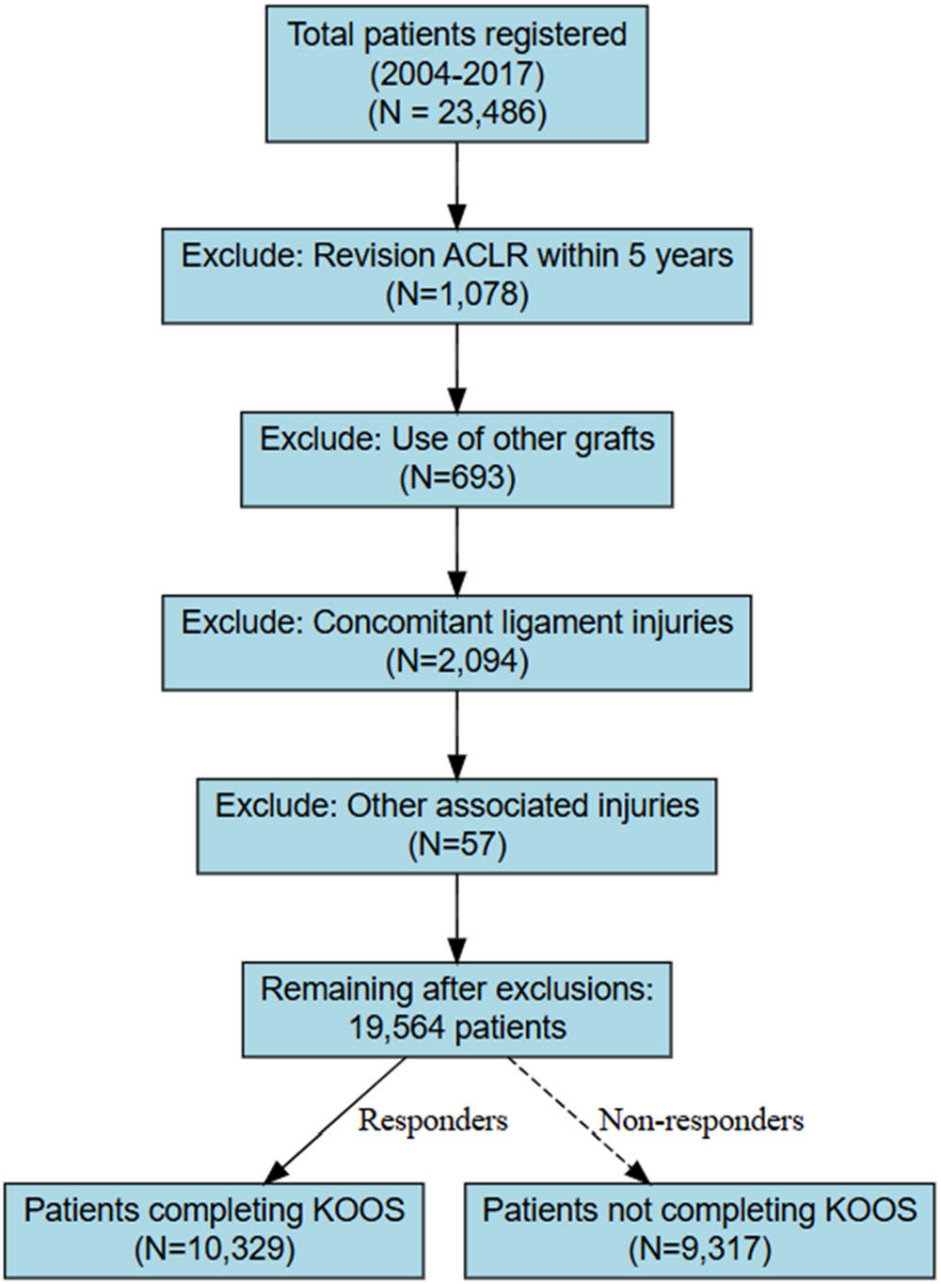
Flowchart of the study population. KOOS, Knee Injury and Osteoarthritis Outcome Score.

From a comprehensive cohort of 23,486 patients registered in the NKLR between 2004 and 2017, rigorous application of inclusion and exclusion criteria yielded a final study population of 10,329 patients (Figure 1), representing one of the most extensive comparative analyses of graft‐specific outcomes in ACL reconstruction.

Baseline demographic and clinical characteristics demonstrated well‐balanced groups with clinically relevant differences (Table 1). The overall mean age at surgery was 29.91 years (SD = 10.96), with a mean time from injury to surgery of 22.78 months (SD = 46.20; median: 8 months; IQR: 4 months). Hamstring autografts were utilised in 6,650 patients (64.4%), while BPTB autografts were selected for 3679 patients (35.6%). Importantly, BPTB grafts were more frequently chosen for younger patients engaged in pivoting sports who underwent surgery within one year of injury, reflecting contemporary surgical decision‐making patterns (Table 1).

**Table 1:** Demographic and clinical characteristics of the total study population and stratified by graft type (*n* = 10,329).

Characteristic	Total (*N* = 10,329)	BPTB graft (*N* = 3679)	Hamstring graft (*N* = 6650)	*p* value
Gender				
Female (%)	50.0	50.8	49.6	0.1
Affected side				
Right knee (%)	50.5	51.6	49.9	0.3
Age at surgery (years)				
Mean (SD)	29.9 (10.9)	29.2 (10.7)	30.3 (11.0)	<0.01
Age ≤ 25 years (%)	47.7	44.5	40.3	<0.01
Time to surgery				
Operated within 12 months (%)	66.7	69.7	65.0	<0.01
Cartilage Injury (%)	23.0	23.7	22.6	0.2
Meniscal Injury (%)	52.3	53.0	51.9	0.3
Pivoting sport at injury (%)	59.0	60.9	57.3	<0.01

*Note*: Continuous variables were analysed using the Mann–Whitney *U* test, and categorical variables were compared with the Pearson chi‐square test. *p*‐Values were calculated accordingly to assess statistical significance.

### Primary outcome analysis: Graft‐specific functional limitations (P1, S4, SP5)

Multivariable logistic regression identified distinct risk profiles for treatment failure across the three primary outcome domains (Table 2). These findings provide the first comprehensive evidence of graft‐specific differences in donor‐site morbidity at 5‐year follow‐up.

**Table 2:** Factors influencing “treatment failure” for primary outcomes (P1, S4, SP5).

Outcome	Variable	Odds ratio (OR)	95% Confidence interval (CI)	*p* value/significance
P1	Hamstrings	1.0	0.9–1.2	0.7
P1	Pivoting sports	1.1	1.0–1.2	0.008
P1	Time to op >12 months	1.1	1.0–1.4	0.01
P1	Age at surgery>25 years	1.5	1.3–1.7	<0.001
P1	Meniscus injury	1.02	0.9–1.1	0.4
P1	Cartilage injury	1.5	1.3–1.7	<0.001
P1	Sex (female)	1.3	1.1–1.4	0.04
S4	Hamstrings	0.9	0.8–1.0	0.049
S4	Pivoting sports	1.0	0.9–1.2	0.218
S4	Time to op <12 months	0.9	0.8–1.1	0.117
S4	Age at surgery>25 years	1.2	1.0–1.3	0.021
S4	Meniscus injury	1.1	1.1–1.2	<0.001
S4	Cartilage injury	1.3	1.1–1.5	0.05
S4	Sex (female)	1.4	1.2–1.6	<0.001
SP5	Hamstrings	0.6	0.5–0.6	<0.001
SP5	Pivoting sport	1.1	1.0–1.2	0.001
SP5	Time to op >12 months	1.2	1.1 ‐ 1.3	<0.001
SP5	Age at surgery>25 years	0.9	0.9–1.1	0.785
SP5	Meniscus injury	1.01	0.9–1.0	0.404
SP5	Cartilage injury	1.3	1.2–1.5	<0.001
SP5	Sex (female)	1.6	1.5–1.8	<0.001

*Note*: Multiple logistic regression analysis was performed to identify factors influencing treatment failure for each outcome. *p*‐Values were calculated accordingly to assess statistical significance

For knee pain frequency (P1), no significant graft‐related differences emerged. However, consistent risk factors for pain included pivoting activity participation (OR = 1.10; 95% CI: 1.03–1.19; *p* = 0.008), delayed surgery >12 months (OR = 1.12; 95% CI: 1.04–1.36; *p* = 0.01), age >25 years (OR = 1.45; 95% CI: 1.28–1.69; *p* < 0.001), cartilage injury (OR = 1.45; 95% CI: 1.26–1.67; *p* < 0.001), and female sex (OR = 1.27; 95% CI: 1.12–1.44; *p* = 0.04).

For extension deficits (S4), hamstring grafts demonstrated a protective effect (OR = 0.86; 95% CI: 0.75–1.00; *p* = 0.049), while established risk factors included age > 25 years (OR = 1.19; 95% CI: 1.03–1.30; *p* = 0.021), meniscal injury (OR = 1.14; 95% CI: 1.09–1.19; *p* < 0.001), cartilage injury (OR = 1.26; 95% CI: 1.07–1.49; *p* = 0.05), and female sex (OR = 1.39; 95% CI: 1.20–1.61; *p* < 0.001).

Most notably, for kneeling difficulty (SP5), hamstring grafts provided substantial protection against functional limitations, reducing failure risk by 43% compared to BPTB grafts (OR = 0.57; 95% CI: 0.51–0.62; *p* < 0.001). Additional risk factors included pivoting sports participation (OR = 1.09; 95% CI: 1.04–1.15; *p* = 0.001), delayed surgery >12 months (OR = 1.21; 95% CI: 1.09–1.33; *p* < 0.001), cartilage injury (OR = 1.33; 95% CI: 1.19–1.47; *p* < 0.001 and female sex (OR = 1.62; 95% CI: 1.47–1.77; *p* < 0.001).

**Figure 2:**
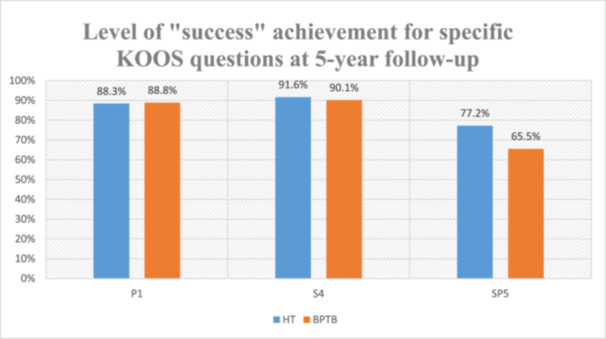
Success percentage for specific KOOS questions at 5‐year follow‐up. BPTB, bone–patellar tendon–bone; HT, hamstrings; KOOS, Knee Injury and Osteoarthritis Outcome Score.

When comparing success rates between grafts, hamstring recipients demonstrated significantly superior outcomes for kneeling function (SP5) (*p* < 0.001), while no significant differences were observed for pain (P1) or extension (S4) (Figure 2).

### PASS achievement

Analysis of clinically meaningful recovery benchmarks revealed graft‐specific differences in functional domains (Table 3). Hamstring grafts conferred a 29% higher likelihood of achieving PASS for Sport/Recreation outcomes (OR 1.29; 95% CI: 1.19–1.41; *p* < 0.001), while no significant difference was observed for QoL outcomes (OR 1.01; 95% CI: 0.92–1.10; *p* = 0.9).

**Table 3:** Comparative overview of predictors for achieving PASS in sport/rec and quality of life outcomes.

Variable	PASS Sport/Recreation (OR, 95% CI, *p*‐value)	PASS QoL (OR, 95% CI, *p* value)
Graft type (hamstring)	1.29 (1.19–1.41, *p* < 0.001)	1.01 (0.92–1.10, *p* = 0.9)
Age at surgery > 25 years	0.93 (0.85–1.01, *p* = 0.1)	1.10 (1.00–1.21, *p* = 0.046)
Time to operation > 12 months	0.84 (0.77–0.91, *p* < 0.001)	0.76 (0.69–0.83, *p* < 0.001)
Pivoting sport	0.93 (0.89–0.98, *p* = 0.004)	0.96 (0.91–1.01, *p* = 0.1)
Meniscus injury	0.99 (0.97–1.02, *p* = 0.78)	0.70 (0.63–0.78, *p* < 0.001)
Cartilage injury	0.70 (0.64–0.78, *p* < 0.001)	0.97 (0.95–0.99, *p* = 0.033)
Sex (female)	0.69 (0.63–0.77, *p* < 0.001)	0.82 (0.75–0.89, *p* < 0.001)

*Note*: Logistic regression identified predictors of achieving Patient‐Acceptable Symptom State (PASS) in Sport/Recreation and Quality of Life (QoL). Odds ratio (OR) indicates the likelihood of success, with a 95% confidence interval (CI) showing the range of possible true ORs. *p*‐Values < 0.05 denote statistically significant predictors.

Consistent predictors of PASS failure across both domains included delayed surgery >12 months (Sport/Recreation: OR 0.84; 95% CI: 0.77–0.91; QoL: OR 0.76; 95% CI: 0.69–0.83; both *p* < 0.001), female sex (Sport/Recreation: OR 0.69; 95% CI: 0.63–0.77; QoL: OR 0.82; 95% CI: 0.75–0.89; both *p* < 0.001) and cartilage injury (Sport/Recreation: OR 0.70; 95% CI: 0.64–0.78; QoL: OR 0.97; 95% CI: 0.95–0.99; both *p* < 0.001).

**Figure 3:**
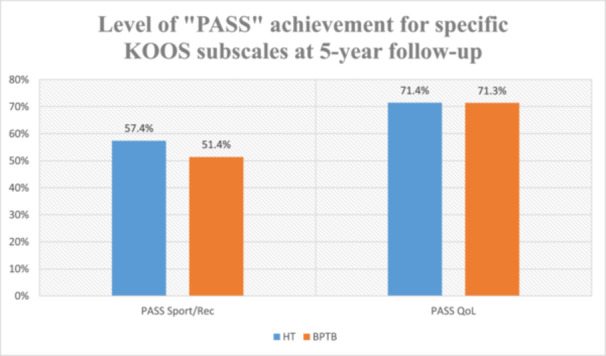
Percentage of patients achieving a PASS for specific KOOS subscales at 5‐year follow‐up. BPTB, bone–patellar tendon–bone; HT, hamstrings; KOOS, Knee Injury and Osteoarthritis Outcome Score; PASS, Patient‐Acceptable Symptom State; QoL, quality of life.

A direct comparison of PASS achievement rates confirmed the superiority of hamstring grafts in Sport/Recreation outcomes (*χ*² = 35.023, *p* < 0.001), with no significant difference in QoL outcomes (*χ*² = 0.009, *p* = 0.9) (Figure 3).

### Absolute KOOS score analysis (Sport/Rec, QoL)

Multiple linear regression analysis of absolute KOOS subscale scores provided additional evidence of graft‐specific benefits (Table 4). Hamstring grafts were associated with modest but significantly higher Sport/Recreation scores (*B* = 2.64; 95% CI: 1.54–3.74; *p* < 0.001), while showing no significant difference in QoL scores (*B* = 0.39; 95% CI: −0.58 to 1.35; *p* = 0.431).

**Table 4:** Summary of multiple linear regression results for 5‐Year KOOS subscale (Sport/Rec and QoL).

Covariate	Effect (Sport/Rec)	*p* value (Sport/Rec)	95% CI (Sport/Rec)	Effect (QoL)	*p* value (QoL)	95% CI (QoL)
Hamstring	2.64	*p* < 0.001	[1.54, 3.74]	0.39	*p* = 0.431	[−0.58, 1.35]
Age at surgery > 25 years	−1.51	*p* = 0.009	[−2.64, −0.37]	1.81	*p* < 0.001	[0.82–2.81]
Time to operation > 12 months	−3.10	*p* < 0.001	[−4.24, −1.96]	−3.85	*p* < 0.001	[−4.85, −2.85]
Pivoting activity	−2.01	*p* < 0.001	[−2.62, −1.40]	−1.06	*p* < 0.001	[−1.59, −0.52]
Meniscus injury	−0.23	*p* = 0.140	[−0.54, 0.08]	−0.26	*p* = 0.057	[−0.54, 0.01]
Cartilage injury	−5.79	*p* < 0.001	[−7.08, −4.49]	−4.77	*p* < 0.001	[−5.89, 3.64]
Sex (female)	−5.17	*p* < 0.001	[−6.25, −4.09]	−2.43	*p* < 0.001	[−3.36, −1.49]

*Note*: Multiple linear regression assessed factors influencing 5‐year KOOS subscale scores for Sport/Recreation and quality of life. Effect sizes indicate the estimated change in scores per covariate; 95% confidence intervals show the range within which the actual effect is likely to lie. *p*‐Values < 0.05 indicate statistically significant predictors.

Abbreviations: CI, confidence interval; KOOS, Knee Injury and Osteoarthritis Outcome Score; QoL, quality of life.

Consistent with previous analyses, female sex emerged as a strong predictor of inferior outcomes across both subscales (Sport/Recreation: *B* = ‐5.17; 95% CI: −6.25 to −4.09; QoL: *B* = −2.43; 95% CI: −3.36 to −1.49; both *p* < 0.001). Similarly, delayed surgery >12 months (Sport/Recreation: *B* = −3.10; QoL: *B* = −3.85; both *p* < 0.001) and cartilage injury (Sport/Recreation: *B* = −5.79; QoL: *B* = −4.77; both *p* < 0.001) were associated with significantly poorer outcomes.

### Responder/non‐responder analysis

Analysis of response patterns demonstrated excellent representativeness of the study cohort. Responders were younger at the time of surgery (27.7 ± 9.6 vs. 30.0 ± 10.9 years, *p* < 0.001) and had a lower BMI (24.8 ± 3.6 vs. 28.9 ± 10.4, *p* < 0.001). Female patients demonstrated higher response rates (62.63% vs. 47.46%, *p* < 0.001), and hamstring graft recipients showed marginally higher response rates compared to BPTB recipients (54.13% vs. 52.68%, *p* = 0.041). In summary, factors such as younger age, female gender, use of hamstring grafts, and a lower BMI were associated with a higher response rate at the 5‐year follow‐up.

## DISCUSSION

### Clinical significance and novel findings

This study represents the most extensive registry‐based analysis examining graft‐specific donor‐site morbidity at 5‐year follow‐up, providing crucial evidence for informed decision‐making in personalised ACL reconstruction. The most clinically significant finding was that hamstring tendon autografts substantially reduced the risk of kneeling difficulties by 43% compared to BPTB grafts (OR 0.57; *p* < 0.001), representing the first comprehensive quantification of this donor‐site morbidity difference at long‐term follow‐up. While overall functional outcomes appeared similar between grafts using conventional measures, detailed analysis of specific KOOS questions and PASS thresholds revealed clinically meaningful differences that directly impact patient counselling and graft selection strategies.

### Graft type and primary outcomes

The investigation of three critical primary outcomes (pain frequency, extension ability, and kneeling function) revealed distinct graft‐specific patterns. The substantial reduction in kneeling difficulties with hamstring grafts aligns with mounting evidence from recent systematic reviews, which demonstrate increased anterior knee pain and kneeling problems associated with BPTB grafts [35]. Zhao et al.'s comprehensive meta‐analysis confirmed significantly higher rates of kneeling pain (OR 1.67; *p* = 0.03) and anterior knee pain (OR 2.90; *p* = 0.002) in BPTB recipients, while Rahardja et al. found that BPTB patients were three times more likely to report severe kneeling difficulty at 2‐year follow‐up [27, 41]. These 5‐year findings extend these observations, demonstrating that this functional limitation persists in the long term, with particular relevance for patients whose occupational or recreational activities require frequent kneeling.

Notably, no significant differences in graft‐related outcomes emerged for pain frequency (P1) or extension deficits (S4) after adjusting for confounding variables. This finding supports longitudinal studies showing that while early postoperative anterior knee pain is more frequent with BPTB grafts, this difference typically diminishes by 2–5 years post‐surgery [14, 21].

However, these results for extension deficits revealed a modest protective effect of hamstring grafts (OR 0.86; *p* = 0.049), suggesting subtle but persistent differences in extensor mechanism function. These findings support contemporary evidence that, while acute donor‐site morbidity may resolve, specific functional limitations can persist in the long term, emphasising the importance of graft selection based on individual patient priorities [41]. The results for knee extension (S4) also align with the findings of Ageberg et al., who reported a similar range of motion across various graft types and no significant differences in knee extension power [1]. However, the authors also found that after three years, the power of the hamstring muscles and the hamstring‐to‐quadriceps ratio were lower in the HT group compared to the BPTB group [1].

### KOOS subscales and PASS

To our knowledge, this study provides the most comprehensive analysis of graft‐specific PASS achievement in ACL reconstruction to date. The 29% higher likelihood of achieving PASS for Sport/Recreation outcomes with hamstring grafts (OR 1.29; *p* < 0.001) represents a clinically meaningful difference that extends beyond traditional outcome measures. This finding contrasts with the limited previous literature, including Hamrin Senorski et al.'s smaller cohort (*n* = 343), which found no graft‐related differences in Sport/Recreation PASS achievement [12]. The discrepancy may reflect our larger sample size, more extended follow‐up period, and different patient demographics, highlighting the importance of registry‐based studies for detecting clinically relevant but modest effect sizes.

The Swedish National ACL Registry and the New Zealand Registry have both analysed the absolute KOOS subscale values for patients undergoing ACLR with either BPTB or HT [27, 33]. They reported that at the two‐year follow‐up, the scores across all KOOS subscales were comparable, indicating that functional outcomes are similar regardless of the graft type [27, 33]. In contrast, the present study suggests that at five years following ACLR, HT is associated with a significantly increased likelihood of achieving higher levels of KOOS in the Sport/Rec subscale. The superior PASS achievement for Sport/Recreation outcomes with hamstring grafts (OR 1.29, *p* < 0.001) likely reflects multiple interconnected mechanisms beyond kneeling ability alone. First, reduced anterior knee pain and kneeling difficulties with hamstring grafts directly affect sports that require frequent knee flexion, such as squatting, lunging, and kneeling, which are fundamental to many athletic activities. BPTB grafts create a patellar tendon defect that leads to patella lowering and increased patellofemoral compression forces during these flexed‐knee positions, potentially resulting in anterior knee pain [39]. Second, injury to the infrapatellar branch of the saphenous nerve during BPTB harvest can lead to persistent anterior knee hypersensitivity, which may limit confidence during dynamic sporting movements [14]. Third, biomechanical studies suggest that altered patellofemoral mechanics following BPTB harvest can affect force transmission during movements common in recreational sports [39]. Our study observed reduced deficits in knee extension function with HT autografts (S4: OR 0.86, *p* = 0.049), which may accumulate to impact sport‐specific performance. Previous literature shows conflicting results: Swedish and New Zealand registries report comparable Sport/Recreation scores at 2 years, whereas our 5‐year data suggest that these differences emerge with longer follow‐up, possibly as patients return to more demanding athletic activities [27, 33, 37]. This nuanced finding has important implications for patient counselling, particularly for athletes who prioritise returning to high‐level sports participation versus recreational activities.

### Patient‐specific risk factors for worse clinical outcomes

Several risk factors associated with inferior clinical and patient‐reported outcomes are correlated with findings in the existing literature. Notably, delays in ACL reconstruction (ACLR), cartilage and meniscus injuries, and female sex are significantly associated with lower post‐operative function scores. Prior studies, such as the one by Senorski et al., demonstrate the adverse effect of concomitant cartilage damage on KOOS outcome after ACLR [13]. Furthermore, a review by Mok et al. identified female gender as a significant risk factor for achieving poorer functional outcomes after ACLR [20]. This was also supported by findings indicating that males had lower odds of not achieving the minimal important change (MIC) for both the Sport/Rec and QoL KOOS subscales one year after ACLR [16]. The observed link between male sex and improved post‐operative outcome after ACLR may be attributed to variations in rehabilitation approaches and psychosocial factors, such as self‐efficacy [16]. Research suggests that males tend to sustain a more optimistic outlook compared to females, which could contribute to these differences in outcomes [17, 23]. Regarding the timing of ACLR, postponing surgery may raise the likelihood of further injuries to both the meniscus and cartilage, especially the medial meniscus, as a result of knee joint instability [40]. Further, previous studies have documented that patients with concurrent cartilage injuries tend to experience more persistent symptoms and pain following ACLR than those without such injuries. This can lead to lower activity levels and diminished quality of life [2]. According to Cristiani et al., delaying ACL reconstruction beyond 12 months is linked to a higher risk of cartilage and meniscal injuries [5]. Postponing the surgery for more than six months increases the likelihood of having persistent 'pre‐reconstruction' knee laxity [5]. To minimise meniscal damage and prevent worsening knee instability, they recommended that ACL reconstruction should be performed within six months of the injury [5].

### Responder/non‐responder analysis

The higher response rate among hamstring recipients (54.13% vs. 52.68%) represents a novel finding that warrants consideration in future registry studies. However, the observed differences in age and sex between responders and non‐responders are consistent with established registry research patterns and do not suggest systematic bias that would compromise study conclusions [29]. However, other studies have typically not found significant differences in BMI between responders and non‐responders [28]. The younger age and lower BMI among responders likely reflect the natural history of ACL reconstruction, where patients with better baseline characteristics may be more motivated to participate in long‐term follow‐up.

### Graft selection considerations: Balancing benefits and limitations

While these findings demonstrate clear advantages of hamstring grafts for kneeling function and Sport/Recreation PASS achievement, BPTB grafts offer essential benefits that warrant consideration in discussions on graft selection. Registry data indicate that revision rates for BPTB grafts are lower, leading to a preference shift since 2013 [24]. BPTB grafts heal faster, typically in about 8 weeks, compared to hamstring grafts, which usually take around 12 weeks [11]. This quicker healing time could allow for a faster rehabilitation process. Second‐look arthroscopies reveal that BPTB grafts demonstrate better maturation and higher return‐to‐sport rates for athletes involved in pivoting sports [26, 37]. Our analysis showed that BPTB grafts are preferred among younger patients and athletes engaged in pivoting sports. While BPTB grafts are ideal for young athletes seeking a rapid return to play, hamstring grafts may be optimal for patients whose occupational or recreational activities involve frequent kneeling or who prioritise minimising anterior knee pain. Future research should identify patient‐specific and injury‐specific predictors that guide optimal graft selection for individual circumstances.

### Strengths and limitations

This study's major strengths include its unprecedented sample size (*n* = 10,329) from a validated national registry, rigorous methodology adhering to STROBE guidelines, and comprehensive 5‐year follow‐up with validated outcome measures. The use of specific KOOS questions targeting donor‐site morbidity and PASS thresholds provides clinically relevant outcomes that directly inform patient counselling. However, several limitations warrant consideration. The retrospective design limits causal inference, and temporal changes in surgical techniques and rehabilitation protocols over the 13‐year study period may influence outcomes. Additionally, the Norwegian population may limit the generalisability of the findings to other healthcare systems or patient populations; however, the large sample size and registry‐based methodology enhance the external validity of the results. The 53% response rate at 5‐year follow‐up aligns with other registry‐based studies but requires careful interpretation. Responders were significantly younger, had lower BMI, were more often female, and were slightly more likely to have received hamstring grafts. These factors may introduce selection bias. The younger age and lower BMI may indicate better baseline functional status, potentially skewing outcome estimates. Additionally, the higher response rate in females, along with evidence that female sex is a risk factor for inferior outcomes (OR 1.62 for kneeling difficulties), suggests that reported complication rates could be conservative. However, the marginally higher response rate among hamstring recipients is unlikely to affect our conclusions significantly, given the large sample size. Overall, these biases likely underestimate donor‐site morbidity, reinforcing our findings of superior kneeling function with hamstring grafts. Future multi‐national registry collaborations could address generalisability concerns while maintaining the methodological rigour demonstrated in this study.

## CONCLUSION

These findings provide clinicians with specific, quantifiable evidence to support individualised graft selection: hamstring grafts significantly reduce kneeling difficulties (43% risk reduction, OR 0.57; *p* < 0.001) and improve Sport/Recreation outcomes (29% higher PASS achievement, OR 1.29; *p* < 0.001), while BPTB grafts offer advantages in revision rates and biomechanical stability documented in previous literature. Patient‐specific factors, including occupational demands, athletic goals, and risk tolerance for donor‐site morbidity versus graft failure, should guide personalised discussions of graft selection.

## AUTHOR CONTRIBUTIONS

All authors contributed to the study conception and design. Material preparation, data collection and analysis were performed by Filip Vuletić, Stein Håkon Låstad Lygre and Andreas Persson. The first draft of the manuscript was written by Filip Vuletić and all authors commented on previous versions of the manuscript. All authors read and approved the final manuscript.

## CONFLICT OF INTEREST STATEMENT

The authors declare no conflicts of interest.

## ETHICS STATEMENT

None declared.

## Data Availability

None declared.
